# Supplementary material for: The Fate of Oxidative Strand Breaks in Mitochondrial DNA
Source: Antioxidants (Basel). 2023 May 12;12(5):1087. doi: 10.3390/antiox12051087 (PMC10215735; doi:10.3390/antiox12051087)
Supplement: Supplementary file 1 [file antioxidants-12-01087-s001.zip › antioxidants-2349390-supplementary.pdf]

Supplemental Figure S1:

Sequencing chromatogram showing the genotype of the POLGexo$^{-/-}$ HEK 293 cells line. The red amino acid code indicates the missense change. The blue amino acid code refers to a silent nucleotide change that was additionally introduced in order to disrupt a protospacer adjacent motif (PAM).

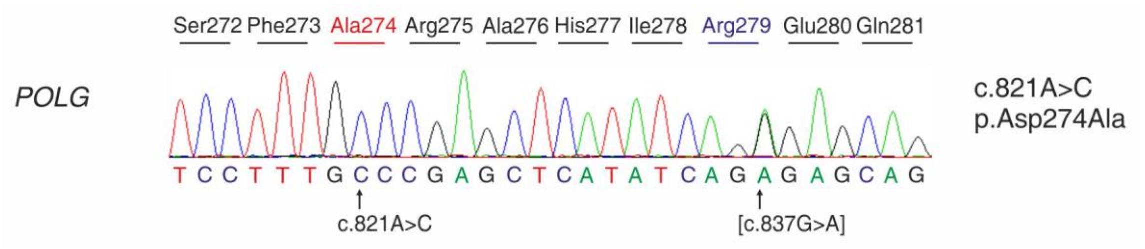

Supplemental Figure S2:

$H_2O_2$ concentration decay in the presence (filled red symbols, red lines) and absence of cells (open symbols, black lines). Upper curves,1 mM $H_2O_2$; lower curves, 0.5 mM $H_2O_2$.

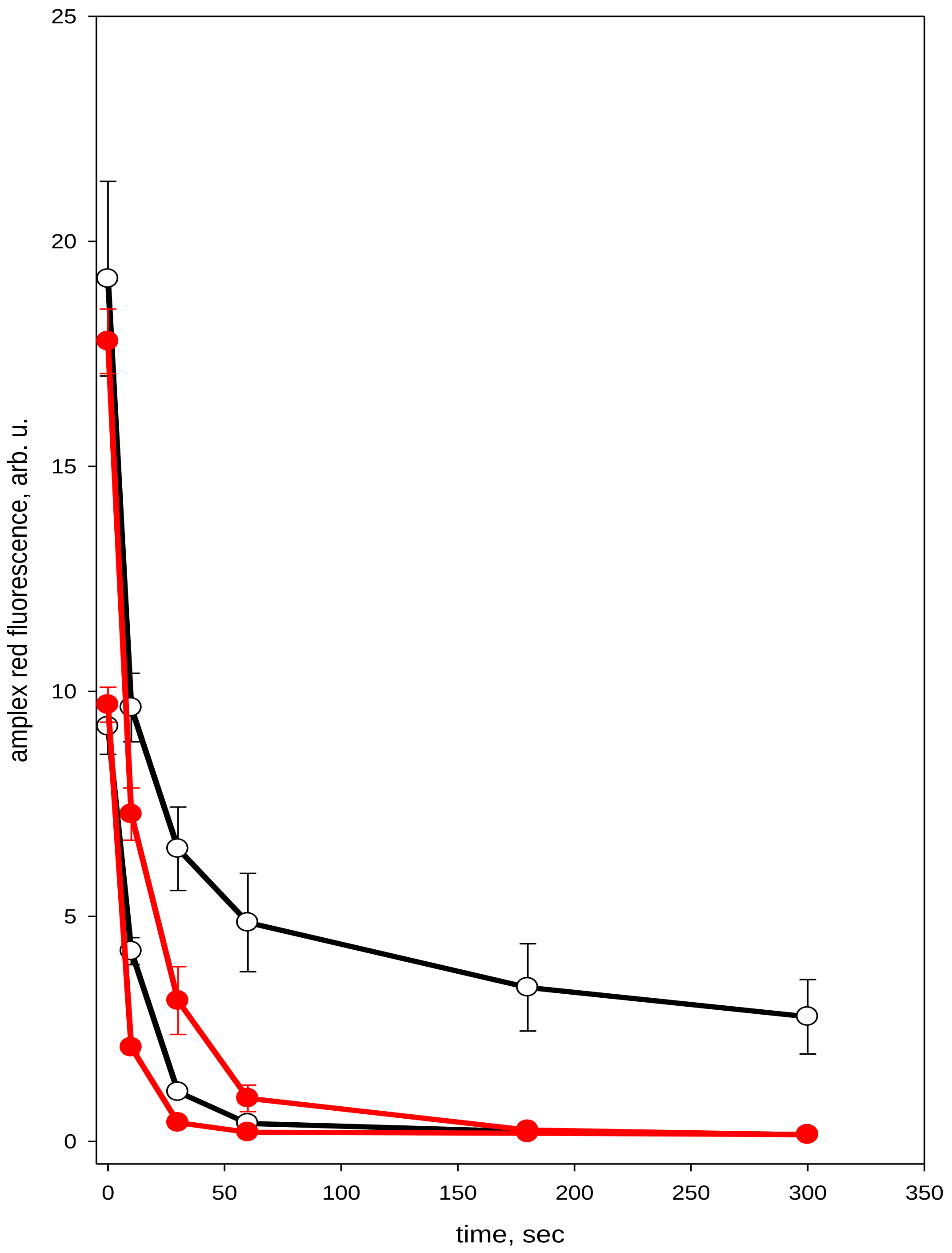


Average amplex red fluorescence data ± SEM determined with three different cell lines are plotted versus time after $H_2O_2$ addition.

Supplemental Figure S3:

Viability of HEK293 cell lines after a 1 mM $H_2O_2$ pulse.

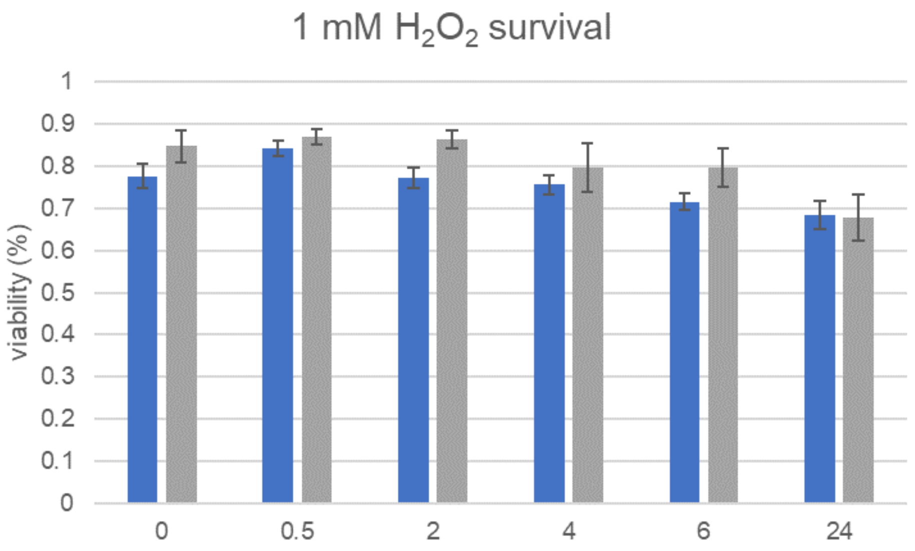


Viability of *n* = 14 wild-type (blue) and *n* = 7 POLGexo$^{-/-}$ (grey) HEK293 cells after a 1 mM $H_2O_2$ pulse. The data are presented as average ± SEM. There is no significant difference in cell survival between wild-type and POLGexo$^{-/-}$ cells. At all time points, the viability remains above 65%, indicating that the cells tolerate the application of 1 mM $H_2O_2$.

Supplemental Figure S4:

BrdU incorporation into wild-type cells in a normal medium and the presence of 1mM $H_2O_2$ (A), and the same blot developed with a MT-ND5 probe (B).

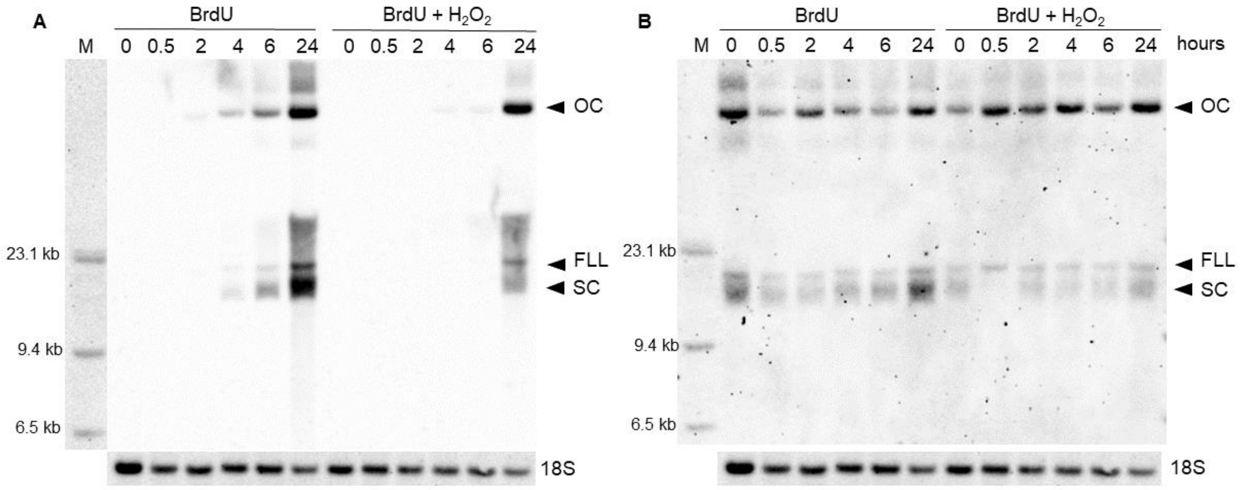


Blotting of time-course BrdU incorporation experiments into wild-type HEK cells in a normal medium and the presence of hydrogen peroxide. The blot has been labeled for the "M" molecular weight marker and the time in hours. The three main conformations of mitochondrial DNA can be seen: open circle (OC), full-length linear (FLL), and the supercoiled (SC). Samples have been digested with *Mlu*I to digest the 18S nuclear DNA as a loading control while leaving the mtDNA undigested. The 18S signal has been developed with a DIG probe for the 18S rRNA. A) BrdU antibody labeled blot. BrdU is incorporated into mtDNA at a slower rate in cells exposed to 1 mM hydrogen peroxide than in a normal medium. The dark smear at 24 hours is a result of BrdU incorporation into nuclear DNA from an incomplete block by aphidicolin. B) The same blot labeled with MT-ND5 DIG-probe for mtDNA. Comparison of blots A and B allows for the confirmation that the bands we see in the BrdU antibody labeled blot are indeed mtDNA. Note the presence of supercoiled mtDNA in all lanes besides the 30 min $H_2O_2$ exposure.
